# Analysis of Air Purification Methods in Operating Rooms of Chinese Hospitals

**DOI:** 10.1155/2020/8278943

**Published:** 2020-02-01

**Authors:** Bingli Zhang, Liuyi Li, Xi Yao, Yuxiu Gong, Yu Zhang, Huai Yang, Weiguang Li, Ling Lin, Yun Yang, Haojun Zhang, Huixue Jia

**Affiliations:** ^1^Peking University First Hospital, Beijing 100034, China; ^2^National Institute of Hospital Administration, Beijing 100034, China; ^3^Guizhou Provincial People's Hospital, Guiyang 550002, China; ^4^Shandong Provincial Hospital, Jinan 250021, China; ^5^Heilongjiang Provincial Center for Disease Control and Prevention, Harbin 150030, China; ^6^Shanxi Dayi Hospital, Shanxi Academy of Medical Sciences, Taiyuan 030001, China; ^7^Gansu Provincial Hospital, Lanzhou 730000, China

## Abstract

This research demonstrates the current use of air purification methods in the operating rooms (ORs) in China. 154 hospitals from 6 provinces were included in this survey to reflect the air purification methods of ORs in 2017. Air cleaning technology (ACT) is used in 124 (80.52%) hospitals. We find that the rates of using grade I, III, or IV clean operating room (COR) in tertiary hospitals are all higher than in lower level hospitals; the rate of using ACT in the ORs is higher, too. In addition, general hospitals have higher rate in using ACT in the ORs than specialized hospitals. The highest rate of using ACT in the ORs is in the eastern region of China. The number of hospitals using ACT, ultraviolet light disinfection, and air sterilizers (such as circulating air UV sterilizer) increased yearly. All grades of CORs can be maintained as required by more than 90% hospitals except grade II COR. In this research, we found air purification methods, especially the ACT, are widely used in hospitals' ORs. However, finding the way to select and use different air purification methods correctly is an urgent problem to be solved next.

## 1. Introduction

Health care-associated infection (HAI) affects the quality of medical care and the safety of health care workers and patients. It also brings a financial burden to the patients and hospitals [1–4]. Surgical-site infection (SSI) is one of the most common HAIs with high morbidity and mortality [5–7]. A study of SSI involving four kinds of surgeries (colorectal surgery, abdominal hysterectomy, femoral neck repair surgery, and vascular surgery) in 29 hospitals in China represented a SSI rate of 1.6% in 2014 [8]; this rate was higher than 0.9% in the US (reported by the National Healthcare Safety Network (NHSN), 2014) [3]. A recent study in the Netherlands also showed that the increased cost of SSI is reaching to €21,569 per case and total costs of DALYs for the three surgery types (colectomy, mastectomy, and total hip arthroplasty) exceeded to €88 million [9].

Researchers report that air microbial contamination in the OR is one of the risk factors for SSI [5, 10]. In 2016, the World Health Organization (WHO) released “Global Guidelines for the Prevention of Surgical Site Infection.” They pointed out the importance of improving the air quality of the OR to prevent SSIs [11]. By properly and rationally using air purification methods, the air quality of ORs can be improved and contribute to reduce the risk of SSI to a certain extent [12, 13]. In 1972, a study on airborne contamination and the deep infection rate published by Charnley showed that, when the ventilation and microbiological performance of the OR were improved, the infection rate would reduce from 7% to 0.5% [14]. Many studies suggested that laminar air flow (LAF) systems and ultraviolet air disinfection in ORs can significantly reduce the SSI rate [15, 16].

In order to regulate the air purification methods in hospitals in China, the Chinese government promulgated the standard “Management Specification of Air Cleaning Technique” in 2012. This standard is aimed for regulating the air purification methods in ORs and usage requirements [17]. The standard has been implemented for more than six years. During this time, many hospitals have undergone significant changes in air purification methods in ORs and new air purification methods have emerged. However, during this period, most of the studies are focusing on the effect of reducing SSIs, only a few of them focused on the status of air purification methods in ORs. Learning the types and composition of air purification methods used in ORs is necessary for the management and the development of air purification methods. In order to understand the current status of air purification methods in China and provide a basis for the management of air purification methods, we conducted this investigation nationwide.

## 2. Materials and Methods

### 2.1. Sampling Methods

A stratified sampling was conducted in six provinces from three regions of China (East, Central, and West). Provincial capital and two prefectural or municipal level cities were selected in each province. We selected 8∼9 hospitals from each city for investigation, including at least one prefectural or municipal level general hospital, one district or county level general hospital, and one specialized hospital. Besides, 1 provincial or ministerial level general hospital was investigated in each province.

### 2.2. Data Collection and Management

The questionnaire was revised and improved by reviewing of the literature and consulting the experts, and then the unified design questionnaire was sent to the surveyed hospitals via a professional questionnaire network. We collected the basic information of hospitals including the hospital level, hospital type, number of beds, annual surgery volume, and air purification methods of ORs in 2017, distribution of various air purification methods in different years, and maintenance of air purification methods. After the reporting of data, the trained staff reviewed the data for errors and inconsistencies and verified data by telephone return visit. In addition, we verified the data reported by hospitals of two provinces in the field investigation. Unverifiable data were processed by “unknown.”

### 2.3. Definitions

#### 2.3.1. Air Cleaning Technology (ACT)

Air cleaning technology is a technique that reduces airborne contaminants concentration by delivering air through a high-efficiency air filter and can be divided into turbulent flow and laminar flow according to the air distribution [13, 18].

#### 2.3.2. Clean Operating Room (COR)

It is the operating room where the total amount of microorganisms and dust particles in the operating environment air are reduced to an allowable level by using ACT. The classification of clean operating room is shown in Table 1 [19].

#### 2.3.3. Chinese Hospital Level

The evaluation system of medical institutions implemented in China divided medical institutions into three levels (tertiary, secondary, and first) through comprehensive evaluation of service quality, technical level, and management level [20]. Some hospitals have not participated in the evaluation yet.

### 2.4. Data Analysis

Categorical variables are expressed as absolute numbers and their relative frequencies. The *χ*^2^ test and Bonferroni method were used to test the differences on rates of using different air purification methods among hospital types, hospital levels, and regions. All statistical analyses were two-sided, and *p* < 0.05 was considered significant. Also, SPSS, version 22.0 was used for data analysis.

## 3. Results

A total of 154 hospitals were investigated, including 12 (7.79%) provincial or ministerial level hospitals, 82 (53.25%) prefectural or municipal level hospitals, and 60 (38.96%) county level hospitals. The characteristics of investigated hospitals, such as hospital level, hospitals type, region, no. of beds, and annual surgery volume, are shown in Table 2.

Among the 154 hospitals, ACT was the most common air purification method in the OR (124 hospitals (80.52%)). Among the 124 hospitals using ACT in the OR, 46 (37.10%) hospitals built only one grade of COR, and 78 (62.90%) built more than one grade of COR (Tables 3 and 4).

The rate of using ACT in the OR of tertiary hospitals was higher than that of lower level hospitals (*p* < 0.05), and we found the rates of using grade I COR, grade III COR, and grade IV COR were higher in tertiary hospitals (*p* < 0.05). The rate of using ACT in the OR in general hospitals was higher than the rate in specialized hospitals (*p* < 0.05), and the difference is mainly caused by the different rate of the grade I COR. Compared with the central and western regions in China, respectively, there were higher rates of using ACT in the OR in the eastern region (*p* < 0.05), and further analysis for different grade of CORs found that the rate of grade I COR was higher in the eastern region than that in other regions (*p* < 0.05) (Tables 5 and 6).

**Figure 1:**
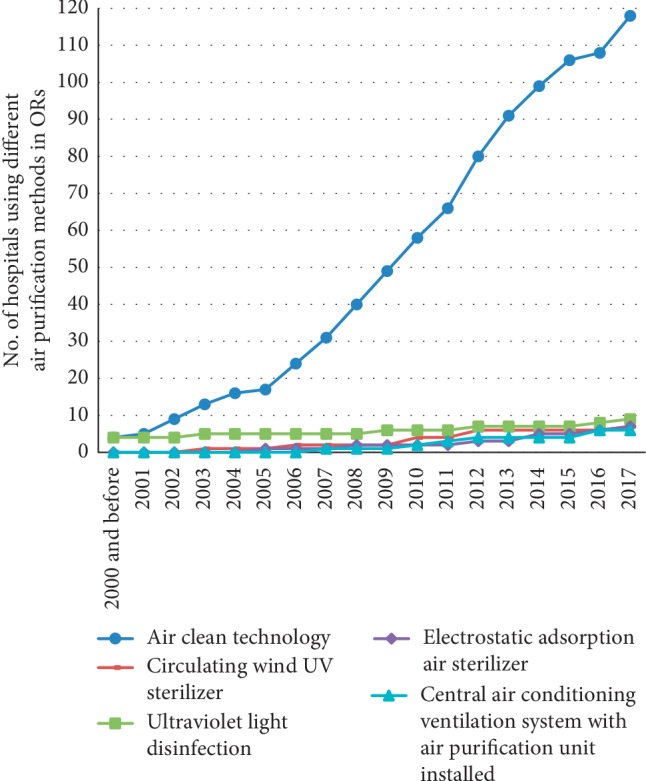
Distribution of various air purification methods in different years. The data are missing for 6 hospitals using ACT in ORs and missing for 1 hospital using circulating wind UV sterilizer in ORs.

From 2001 to 2017, the number of hospitals using ACT, ultraviolet light disinfection, circulating air UV air sterilizer, electrostatic adsorption air sterilizer, and central air conditioning system with air purification device in ORs gradually increased year by year. Among these air purification methods, the number of hospitals using ACT in ORs always occupied top place every year from 2000 to 2017 and increased from 4 (2.60%) in 2000 to 118 (76.62%) in 2017 (Figure 1).

In more than 90% hospitals, grade I CORs, grade III CORs, and grade IV CORs can meet the requirement, while three of the four hospitals can maintain grade II CORs as required. All of the surveyed hospitals can maintain circulating wind UV sterilizer, ultraviolet light disinfection, electrostatic adsorption air sterilizer, and central air conditioning ventilation system with air purification device and mechanical ventilation as required. (Table 7).

## 4. Discussion

Since the first promulgation of the Architectural Technical Code for Hospital Clean Operating Department (GB50333) [19] in 2002, the number of hospitals with CORs has increased significantly in China. Provincial or municipal hospitals and even county level hospitals have begun to establish large-scale, high-standard CORs [21, 22]. This survey found, as of 2017, more than 80% of the hospitals have adopted ACT. Among them, more than 50% of hospitals have built the grade I COR and grade III COR, and most of hospitals with CORs are concentrated in economically developed eastern region. It can be seen that the ACT has become the most widely used air purification method in ORs in China nowadays. And, the number of hospitals using ACT in ORs has increased rapidly year by year, especially the grade I COR and the grade III COR. Standard drafter Jinming Shen once said that the misinterpretation for the norms is one of the reasons for accelerating the construction of CORs. He stressed that the purpose of formulating Architectural Technical Code for Hospital Clean Operating Department was to regulate the construction of the COR rather than to cancel the general OR [23]. In this survey, only four hospitals used the grade II COR, which were much less than the number of hospitals with other grades CORs. Owing to the standard Architectural Technical Code for Hospital Clean Operating Department (GB50333) [15], the requirements for the main technical indicators, basic equipment, layout, indoor decoration, air cleaning and conditioning system, etc. of the grade I COR and those of the grade II COR are almost identical; therefore, many hospitals preferred to build grade I CORs with higher cleanliness level.

ACTs have been widely used in ORs of China, but the effect of ACT on preventing SSIs, especially the LAF, has been controversial [11, 24–26]. In 2016, the WHO released the “Global Guidelines for The Prevention of Surgical Site Infection” [11], the guidelines recommend that “the panel suggests that laminar airflow ventilation systems should not be used to reduce the risk of SSI for patients undergoing total arthroplasty surgery,” but it also mentioned that because the data used in the study were not specifically designed for LAF effects on SSIs, it may be affected by factors such as the number of hospitals, surgeons, characteristics, or implementation of patients admitted. And, the guidelines also mentioned that the single studies found that LAF has different effects on the risk of SSI for different types of surgeries.

In addition, there are also differences in the recommended cleanliness level of the OR for different types of surgeries among countries. In the United States, the American Society of Heating, Refrigerating and Air-Conditioning Engineers (ASHRAE) standard 170-2017 “Ventilation of Health Care Facilities” [27] mentioned that high-risk special operating room is recommended for large-scale surgery requiring general or large-area local anesthesia and vital function maintenance equipment, which is equivalent to grade I COR in China. The standard Architectural Technical Code for Hospital Clean Operating Department (GB50333) [15] in China recommended grade I COR for prosthesis, implantation, some large organ transplants, and surgery with SSI which will directly endanger life and quality of life.

At last, the construction costs on CORs and the maintenance costs of LAF are extremely high. A study in Italy concluded that construction costs of CORs have increased by 24% and annual operating costs have increased by 36% [28]. Domestic research indicated the annual operating cost of a grade I COR can reach more than 100,000 yuan [21, 29, 30]. The construction and maintenance of CORs can increase the national medical expenditure, and the unqualified construction and inadequate maintenance of CORs will make the air cleaning equipment a source of airborne microbial contamination [31, 32], and the low-price bidding policy in China further affected the construction quality of CORs and maintenance [33]. Faced with these questions, should the hospital continue to build CORs and how to properly use ACT in ORs? Hu [33] believed that hospitals should establish CORs according to their own scale, mission requirements, nature, etc. A study has shown the general OR with the proper air purification method can also effectively control the number of bacterial colonies and dust in the air [28]. There have been rules for the construction of general ORs in China. The standard GB15982-2012 Hygienic Standard for Disinfection in Hospitals [34] defined clearly the environmental sanitation requirements of the general OR; the standard GB51039-2014 Code for Design of General Hospital [35] provided relevant regulations for the architectural design and heating, ventilation and air conditioning systems of the general OR; the standard WS/T368-2012 Management Specification of Air Cleaning Technique in Hospitals [17] had listed the air purification methods available for the general OR.

With the development of science and technology, new air purification methods are constantly appearing. The survey found that, in addition to ACT, the number of hospitals using circulating air UV sterilizers and electrostatic adsorption air sterilizers in ORs has been slowly increased year by year. Research has shown that using the dynamic air sterilizer in the OR can reduce the impact of personnel activity on air quality to a certain extent [36]. However, various temperature and humidity in different hospitals caused by the vast territory of China and the different density of personnel in different hospitals lead to that the disinfection effect of one air purification method in different hospitals is diverse. The standard WS/T648-2019 General Hygienic Requirements for Disinfecting Machine [37] issued by Chinese government on March 1, 2019, made requirements for the disinfection effect of air sterilizers and further standardized the sanitary requirements for air sterilizers. Nevertheless, the air disinfection effect of one air purification method in various situations remains to be further studied. At the same time, we found ultraviolet light disinfection is still an important air disinfection method in ORs. Ultraviolet light disinfection has been proven to reduce the level of contamination in ORs and thereby preventing the SSI, but there are issues such as the frequency, amount, and locations for ultraviolet light disinfection needed to be further researched [16]. The survey also found that the use rate of circulating air UV sterilizers has gradually increased to 5.19% in 2017, which was close to that of ultraviolet light disinfection (5.84%). Studies have shown that there was no significant difference in the air disinfection effect between two purification methods under static conditions, but the total amount of microorganisms in the OR is rapidly increased under dynamic conditions after ultraviolet light disinfection [38, 39]. As mentioned above, air disinfection effects of different air purification methods are affected by a lot of factors, so further research is needed.

There are still many doubts about the selection of air purification methods. For example, there is no clear standard for situations requiring the construction of a COR, no rules for surgery type which can be operated in a general OR, and so on. All of the above issues require further research to provide scientific evidence-based evidence for the formulation and revision of national policies. Last but not the least, using air purification methods is just one of the ways to improve the air quality of the ORs. Strengthening the comprehensive management of the OR also plays an important role in improving and maintaining the quality of the air in the OR, including the development of the corresponding management system, controlling the number and the state of the personnel in ORs, the good management between surgeries, controlling the times and time of opening the surgical door, and cleaning and disinfection of the environment. [40–43]. Therefore, we should take comprehensive measures to improve the cleanliness of the OR and reduce the risk of SSI.

## 5. Conclusion

The number of hospitals using ACT in ORs has been increasing year by year. The rate of using different grades of CORs varies according to hospital level, region, and hospital type. Other air purification methods, including ultraviolet light disinfection, circulating wind UV sterilizer, and electrostatic adsorption air sterilizer also widely used in hospitals' ORs. How to correctly select and use different air purification methods is an urgent problem to be solved.

## Figures and Tables

**Table 1:** Classification of clean operating room.

Grade	Airborne bacterial concentration (at rest)	
	Operating zone (cfu/m^3^)	Surrounding zone (cfu/m^3^)
I	5	10
II	25	50
III	75	150
IV	175	

**Table 2:** Characteristics of surveyed hospitals.

Characteristics	No. of hospitals	Proportion (%)
Hospital level		
Tertiary	91	59.09
Secondary and lower	63	40.91
Hospital type		
General	117	75.97
Specialized	37	24.03
Region		
East	53	34.41
Central	50	32.47
West	51	33.12
No. of beds		
<400 beds	38	24.67
400∼899 beds	51	33.12
>900 beds	65	42.21
Annual surgery volume		
<2000	39	25.32
2000∼	47	30.52
6000∼	68	44.16
Total	154	100.00

**Table 3:** Air purification methods in operating rooms of surveyed hospitals.

Air purification methods	No. of hospitals	Proportion (%)
ACT	124	80.52
Circulating wind UV sterilizer	7	4.54
Electrostatic adsorption air sterilizer	6	3.90
Central air conditioning system with air purification device	6	3.90
Ultraviolet light disinfection	6	3.90
Ultraviolet light disinfection and air sterilizer^*∗*^	3	1.94
Mechanical ventilation	2	1.30
Total	154	100.00

^*∗*^Air sterilizer included electrostatic adsorption air sterilizer, circulating wind UV sterilizer, and plasma air sterilizer.

**Table 4:** Different grade clean operating rooms of surveyed hospitals.

Different grades of COR	No. of hospitals	Proportion (%)
I and III	50	40.32
III	24	19.35
I	19	15.32
I, III, and IV	17	13.71
III and IV	7	5.65
IV	3	2.42
I, II, and III	2	1.61
II and III	1	0.81
I, II, III, and IV	1	0.81
Total	124	100.00

**Table 5:** Analysis of air clean technology in operating rooms of surveyed hospitals.

Characteristics	Total no. of hospitals	No. of hospitals using ACT in ORs (%)	*χ * ^*2*^	*p* value
Hospital level			**13.04**	**<0.01**
Tertiary	91	82 (90.11)		
Secondary and lower	63	42 (66.67)		
Hospital type			**7.61**	**0.01**
General	117	100 (85.47)		
Specialized	37	24 (64.86)		
Region			**7.34**	**0.03**
East	53	49 (92.45)		
Central	50	37 (74.00)		
West	51	38 (74.51)		

**Table 6:** Analysis of different grade clean operating room in surveyed hospitals.

Characteristics	No. of hospitals	No. of hospitals using the COR I (%)	*χ* ^2^	*p* value	No. of hospitals using the COR III (%)	*χ* ^2^	*p* value	No. of hospitals using the COR IV (%)	*χ* ^2^	*p* value
Hospital level			**9.75**	**<0.01**		**11.37**	**<0.01**		**4.76**	**0.03**
Tertiary	91	62 (68.13)			70 (76.92)			23 (25.27)		
Secondary and lower	63	27 (42.86)			32 (50.79)			7 (11.11)		
Hospital type			**23.36**	**<0.01**		0.36	0.55		1.11	0.29
General	117	80 (68.38)			79 (67.52)			25 (21.37)		
Specialized	37	9 (24.32)			23 (62.16)			5 (13.51)		
Region			**6.42**	**<0.05**		0.47	0.79		1.36	0.51
East	53	38 (71.70)			37 (69.81)			13 (24.53)		
Central	50	25 (50.00)			32 (64.00)			8 (16.00)		
West	51	26 (50.98)			33 (64.71)			9 (17.65)		

**Table 7:** Analysis of the maintenance of different air purification methods in surveyed hospitals' ORs.

Air purification methods	No. of hospitals	No. of hospitals with clean maintenance (%)	No. of hospitals replacing key components as required (%)
Grade I of ACT	89	86 (96.63)	85 (95.51)
Grade II of ACT	4	3 (75.00)	3 (75.00)
Grade III of ACT	102	97 (95.10)	99 (97.06)
Grade IV of ACT	30	29 (96.67)	29 (96.67)
Ultraviolet light disinfection	9	9 (100.00)	9 (100.00)
Circulating wind UV sterilizer	8	8 (100.00)	8 (100.00)
Electrostatic adsorption air sterilizer	7	7 (100.00)	7 (100.00)
Central air conditioning ventilation system with air purification device	6	6 (100.00)	6 (100.00)
Mechanical ventilation	2	2 (100.00)	2 (100.00)

## Data Availability

The data used to support the findings of this study are included within the article.

## References

[1] World Health Organization (2011). *Report on the Burden of Endemic Health Care-Associated Infection Worldwide. A Systematic Review of the Literature*.

[2] European Centre for Disease Prevention and Control (2013). *Point Prevalence Survey of Health-Care Associated Infections and Antimicrobial Use in European Acute Care Hospitals*.

[3] National Center for Emerging and Zoonotic Infectious Diseases, Centers for Disease Control and Prevention (2016). *National and State Healthcare-Associated Infections Progress Report*.

[4] Ling M. L., Apisarnthanarak A., Madriaga G. (2015). The burden of healthcare-associated infections in Southeast Asia: a systematic literature review and meta-analysis. *Clinical Infectious Diseases*.

[5] de Lissovoy G., Fraeman K., Hutchins V., Murphy D., Song D., Vaughn B. B. (2009). Surgical site infection: incidence and impact on hospital utilization and treatment costs. *American Journal of Infection Control*.

[6] Leaper D. J., van Goor H., Reilly J. (2004). Surgical site infection—a European perspective of incidence and economic burden. *International Wound Journal*.

[7] Lamarsalle L., Hunt B., Schauf M., Szwarcensztein K., Valentine W. J. (2013). Evaluating the clinical and economic burden of healthcare-associated infections during hospitalization for surgery in France. *Epidemiology and Infection*.

[8] Zhang Y., Liu S. N., Li L. Y. (2015). Multicenter study on targeted monitoring of surgical site infection and risk factors. *Chinese Journal of Infection Control*.

[9] Koek M. B. G., van der Kooi T. I. I., Stigter F. C. A. (2019). Burden of surgical site infections in the Netherlands: cost analyses and disability adjusted life years. *Journal of Hospital Infection*.

[10] Lidwell O. M., Lowbury E. J. L., Whyte W., Blowers R., Stanley S. J., Lowe D. (1983). Airborne contamination of wounds in joint replacement operations: the relationship to sepsis rates. *Journal of Hospital Infection*.

[11] World Health Organization (WHO) (2016). *Global Guide to Prevention of Surgical Site Infection (SSI)*.

[12] Luciano J. R. (1984). New concept in French hospital operating room HVAC systems. *ASHRAE Journal*.

[13] Chow T. T., Yang X. Y. (2004). Ventilation performance in operating theatres against airborne infection: review of research activities and practical guidance. *Journal of Hospital Infection*.

[14] Charnley J. (1972). Section II general orthopaedics postoperative infection after total hip replacement with special reference to air contamination in the operating room. *Clinical Orthopaedics and Related Research*.

[15] Salvati E. A., Robinson R. P., Zeno S. M., Koslin B. L., Brause B. D., Wilson P. D. (1982). Infection rates after 3175 total hip and total knee replacements performed with and without a horizontal unidirectional filtered air-flow system. *The Journal of Bone & Joint Surgery*.

[16] Curtis G. L., Faour M., Jawad M., Klika A. K., Barsoum W. K., Higuera C. A. (2018). Reduction of particles in the operating room using ultraviolet air disinfection and recirculation units. *The Journal of Arthroplasty*.

[17] Ministry of Health of the People’s Republic of China (2013). *Management Specification of Air Cleaning Technique in Hospitals*.

[18] Iudicello S., Fadda A. (2013). A road map to a comprehensive regulation on ventilation technology for operating rooms. *Infection Control & Hospital Epidemiology*.

[19] Ministry of Housing and Urban-Rural Development of the People’s Republic of China (2013). *Architectural Technical Code for Hospital Clean Operating Department*.

[20] The State Council of the People’s Republic of China (2016). *Medical Institution Management Regulations*.

[21] Xu J. S. (2005). Thinking about the application standard and operating cost of hospital clean operating room. *China Health Quality Management*.

[22] Gan Y. J., Lu Y. Y. (2018). Research progress on air clean technology and prevention of surgical site infection. *Chinese Hospital Construction and Equipment*.

[23] Shen J. M., Yu W. G. (2007). Dispel the misunderstandings about architectural technical code for hospital clean operating department. *Chinese Hospital Construction and Equipment*.

[24] Anderson D. J., Podgorny K., Berríos-Torres S. I. (2014). Strategies to prevent surgical site infections in acute care hospitals: 2014 update. *Infection Control & Hospital Epidemiology*.

[25] Gastmeier P., Breier A.-C., Brandt C. (2012). Influence of laminar airflow on prosthetic joint infections: a systematic review. *Journal of Hospital Infection*.

[26] Ling X. L., Ping L. Y. (2013). Effect of laminar flow operating rooms on prevention of surgical infection. *Chinese Journal of Nosocomial*.

[27] ASHRAE (2017). *Ventilation of Health Care Facilities*.

[28] Cacciari P., Giannoni R., Marcelli E. (2004). Cost evaluation of a ventilation system for operating theatre: an ultraclean design versus a conventional one. *Annali di Igiene: Medicina Preventiva e di Comunità*.

[29] Liang X. L., Jia Y. F. (2015). Air quality monitoring and cost estimation in clean operating room and reasonable allocation between operations. *Health Vocational Education*.

[30] Zhao A. M., Zhang Y. (2006). Discussion building economic clean operating room. *Contamination Control & Air-Conditioning Technology*.

[31] Liu X. (2017). Application status of laminar flow ventilation system in operating rooms worldwide. *Chinese Journal of Nosocomial*.

[32] Hu G. Q., Li Y., Gao X. S. (2018). Current status of use of clean operating rooms in 2359 Chinese hospitals. *Chinese Journal of Nosocomial*.

[33] Hu G. Q. (2017). *New Developments in New Standards for Hospital Disinfection and Nosocomial Infection Control*.

[34] General Administration of Quality Supervision, Inspection and Quarantine of the People’s Republic of China (2012). *Hygienic Standard for Disinfection in Hospitals*.

[35] Ministry of Housing and Urban-Rural Development of the People’s Republic of China (2014). *Code for Design of General Hospital*.

[36] Wang L. P. (2010). The clinical observation on the air disinfection effect of dynamic sterilizer in operating room. *Medical Information*.

[37] National Health Commission of the People’s Republic of China (2019). *General Hygienic Requirement for Air Disinfecting Machine*.

[38] Wu M.-h., Wei T.-h. (2015). Air disinfection effect on ultraviolet lamp and circulating wind UV disinfector on stomatology operation room. *Chinese Journal of Disinfection*.

[39] Wu X. T. (2004). Comparison of air disinfection effect between circulating air UV disinfection machine and ultraviolet lamp. *Chinese Journal of Nosocomial*.

[40] Maini L. (2011). Clean operating rooms for optimizing surgical outcome. *Journal of Clinical Orthopaedics and Trauma*.

[41] Lydon G. P., Ingham D. B., Mourshed M. M. (2014). Ultra clean ventilation system performance relating to airborne infections in operating theatres using CFD modelling. *Building Simulation*.

[42] Dancer S. J. (2014). Controlling hospital-acquired infection: focus on the role of the environment and new technologies for decontamination. *Clinical Microbiology Reviews*.

[43] Schulster L., Chinn R. (2003). Guidelines for environmental infection control in health-care facilities: recommendations of CDC and the healthcare infection control practices advisory committee (HICPAC).

